# Tracheal epithelium cell volume responses to hyperosmolar, isosmolar and hypoosmolar solutions: relation to epithelium-derived relaxing factor (EpDRF) effects

**DOI:** 10.3389/fphys.2013.00287

**Published:** 2013-10-11

**Authors:** Jeffrey S. Fedan, Janet A. Thompson, U. Burcin Ismailoglu, Yi Jing

**Affiliations:** Pathology and Physiology Research Branch, National Institute for Occupational Safety and HealthMorgantown, WV, USA

**Keywords:** exercise asthma, epithelium-derived relaxing factor, cell volume

## Abstract

In asthmatic patients, inhalation of hyperosmolar saline or D-mannitol (D-M) elicits bronchoconstriction, but in healthy subjects exercise causes bronchodilation. Hyperventilation causes drying of airway surface liquid (ASL) and increases its osmolarity. Hyperosmolar challenge of airway epithelium releases epithelium-derived relaxing factor (EpDRF), which relaxes the airway smooth muscle. This pathway could be involved in exercise-induced bronchodilation. Little is known of ASL hyperosmolarity effects on epithelial function. We investigated the effects of osmolar challenge maneuvers on dispersed and adherent guinea-pig tracheal epithelial cells to examine the hypothesis that EpDRF-mediated relaxation is associated with epithelial cell shrinkage. Enzymatically-dispersed cells shrank when challenged with ≥10 mOsM added D-M, urea or NaCl with a concentration-dependence that mimics relaxation of the of isolated perfused tracheas (IPT). Cells shrank when incubated in isosmolar N-methyl-D-glucamine (NMDG) chloride, Na gluconate (Glu), NMDG-Glu, K-Glu and K_2_SO_4_, and swelled in isosmolar KBr and KCl. However, isosmolar challenge is not a strong stimulus of relaxation in IPTs. In previous studies amiloride and 4,4′-diisothiocyano-2,2′-stilbenedisulfonic acid (DIDS) inhibited relaxation of IPT to hyperosmolar challenge, but had little effect on shrinkage of dispersed cells. Confocal microscopy in tracheal segments showed that adherent epithelium is refractory to low hyperosmolar concentrations that induce dispersed cell shrinkage and relaxation of IPT. Except for gadolinium and erythro-9-(2-hydroxy-3-nonyl)adenine (EHNA), actin and microtubule inhibitors and membrane permeabilizing agents did not affect on ion transport by adherent epithelium or shrinkage responses of dispersed cells. Our studies dissociate relaxation of IPT from cell shrinkage after hyperosmolar challenge of airway epithelium.

## Introduction

In healthy subjects, bronchodilation accompanies exercise (Gelb et al., 1985; Silverman et al., 2005). Exercise leads to evaporative water loss, dehydration of the airway surface liquid (ASL), and increases ASL osmolarity (Freed and Davis, 1999; Anderson and Daviskas, 2000; Anderson, 2010, 2012). In asthmatic patients, exercise may precipitate bronchoconstriction (exercise-induced bronchoconstriction, EIB) (Weiler et al., 2010; Anderson and Kippelen, 2012; Hallstrand, 2012). EIB is attenuated by leukotriene modifier drugs and glucocorticoids (Kemp, 2009). Asthmatic patients who experience EIB exhibit dysfunction in a compensatory Na^+^-absorptive pathway that regulates ASL (Schmitt et al., 2011). What accounts for the phenotype shift from bronchodilation during exercise to EIB is unknown.

The effect of elevations in ASL osmolarity on respiratory epithelium physiology has been studied on a limited basis and is not understood. Willumsen et al. (1994) using cultured human nasal epithelium reported that the application of D-mannitol (D-M) or NaCl to create hyperosmolar conditions (50–430 mOsM) at the apical surface resulted in decreased thickness of the epithelium and alterations in Na^+^, Cl^−^, and K^+^ transport.

Inhalation of hyperosmotic saline or D-M aerosols also elicits pulmonary obstruction in asthmatic patients (Holzer et al., 2003; de Meer et al., 2004) that is thought to result from elevation in ASL osmolarity and involve EIB mediators. Inhaled hyperosmotic saline and D-M aerosols are efficacious agents for identifying bronchial hyperreactivity in asthmatic patients (Brannan et al., 2005; Anderson, 2010; Wood et al., 2010). Inhalation of hyperosmotic solutions and D-M by cystic fibrosis patients reduces exacerbations, and improves pulmonary function and hydration of sputum (Elkins et al., 2006; Daviskas et al., 2010; Aitken et al., 2012). Hogg and Eggleston (1984) asked, “Is asthma an epithelial disease?” in relation to the effects of inhaled isosmolar and non-isosmolar aerosols in asthmatic patients. A corollary question, “what are the effects of raised osmolarity of the ASL on airway function?,” has not been addressed and, therefore, has been investigated in our laboratory. In the guinea-pig isolated, perfused trachea (IPT) preparation, hyperosmolar challenge of the epithelium induces relaxation of the airway smooth muscle (Munakata et al., 1988; Fedan et al., 1999, 2004a,b; Johnston et al., 2004; Wu et al., 2004; Jing et al., 2008a) that is inhibited by the Na^+^ channel blocker, amiloride, and the Cl^−^ channel blockers, 4, 4′-diisothiocyano-2, 2′-stilbenedisulfonic acid (DIDS) and 5-nitro-2-(3-phenylpropylamino) benzoic acid (NPPB). Ionic and non-ionic, permeant and impermeant osmolytes have similar relaxant potencies (~9–25 mOsM). Relaxation is elicited with as little as 3–5 mOsM increments. The osmotic relaxant effect is very powerful. For example, 120 mM KCl added to the serosal surface of the trachea elicits depolarization of the smooth muscle and contraction, but, applied to the lumen of the trachea, it causes relaxation, thereby overwhelming any effect that KCl might have had on the muscle after diffusing across the epithelium. The relaxations are dependent upon the presence of the epithelium and mediated *via* the release of epithelium-derived relaxing factor (EpDRF). EpDRF resembles, in part, carbon monoxide; it is not nitric oxide or a prostanoid. p38 is involved in EpDRF-mediated relaxation (Jing et al., 2008a). Relaxation responses are not inhibited by cytoskeleton/microtubule-interfering agents. EpDRF release occurs in response to incremental increases in osmolarity rather than sensing of the absolute osmolarity. Functional evidence was obtained to suggest that the EpDRF release initiated by hyperosmolar challenge is unrelated to cell shrinkage; this evidence was indirect. Hyperosmolar challenge evokes electrophysiological responses that are complex, osmolyte-specific and concentration-dependent, polarized across the epithelium and involve activation of JNK, PKC and phosphatases (Wu et al., 2004; Jing et al., 2008b). The osmosensor which triggers these responses is undescribed.

Lipopolysaccharide treatment *in vivo* (Dodrill and Fedan, 2010) or exposure to cytokines *in vitro* (Ismailoglu et al., 2009) potentiated hyperosmolarity-induced relaxation. Lipopolysaccharide treatment *in vivo* also increased transepithelial potential difference (*V*_*t*_) and potentiated depolarization responses to elevations in osmolarity (Johnston et al., 2004). These findings suggest that the EpDRF system is regulated dynamically in these models and might occur in lung diseases beyond asthma.

Hyperosmolar and hypoosmolar solutions applied to airway epithelium also induce vasodilation and vasoconstriction, respectively, of submucosal blood vessels (Prazma et al., 1994), implying that the epithelium is involved in regulation of blood flow and that this axis is modulated by ASL tonicity.

Mammalian cells shrink when exposed to a hyperosmolar environment (Strange, 1994; Wehner et al., 2003; Lang, 2006; Hoffmann et al., 2009). Previously, our hypothesis that EpDRF release is not attributable to epithelial bioelectric events or cell shrinkage was supported indirectly by functional experiments in the IPT using osmolar maneuvers known to affect volume in other cell types. In the present investigation we evaluated this hypothesis further by measuring cell volume responses of dispersed and adherent tracheal epithelial cells to solutions of varying composition and osmolarity, and examined the effects of blockers of ion transport, cytoskeleton/microtubules reorganization, signaling, mediator formation, and membrane permeabilizing agents. We utilized experimental conditions and protocols similar to those that had been employed in IPT experiments to enable comparisons between the two investigative approaches. Our findings dissociate cell shrinkage from EpDRF release in response to hyperosmolar challenge.

## Materials and methods

### Animals

These studies were conducted in facilities accredited fully by the Association for the Assessment and Accreditation of Laboratory Animal Care International and the research protocol was approved by the Institutional Animal Care and Use Committee. Male Hartley guinea pigs (Crl:Ha 600–700 g) from Charles River Laboratories (Wilmington, MA), monitored free of endogenous viral pathogens, parasites, and bacteria, were used in all experiments. The animals were acclimated before use and were housed in filtered ventilated cages on Alpha-Dri virgin cellulose chips and hardwood Beta chips as bedding, provided HEPA-filtered air, Teklad 7906 diet and tap water *ad libitum*, under controlled light cycle (12 h light) and temperature (22–25°C) conditions.

### Preparation of epithelial cell suspensions

**Figure 1:**
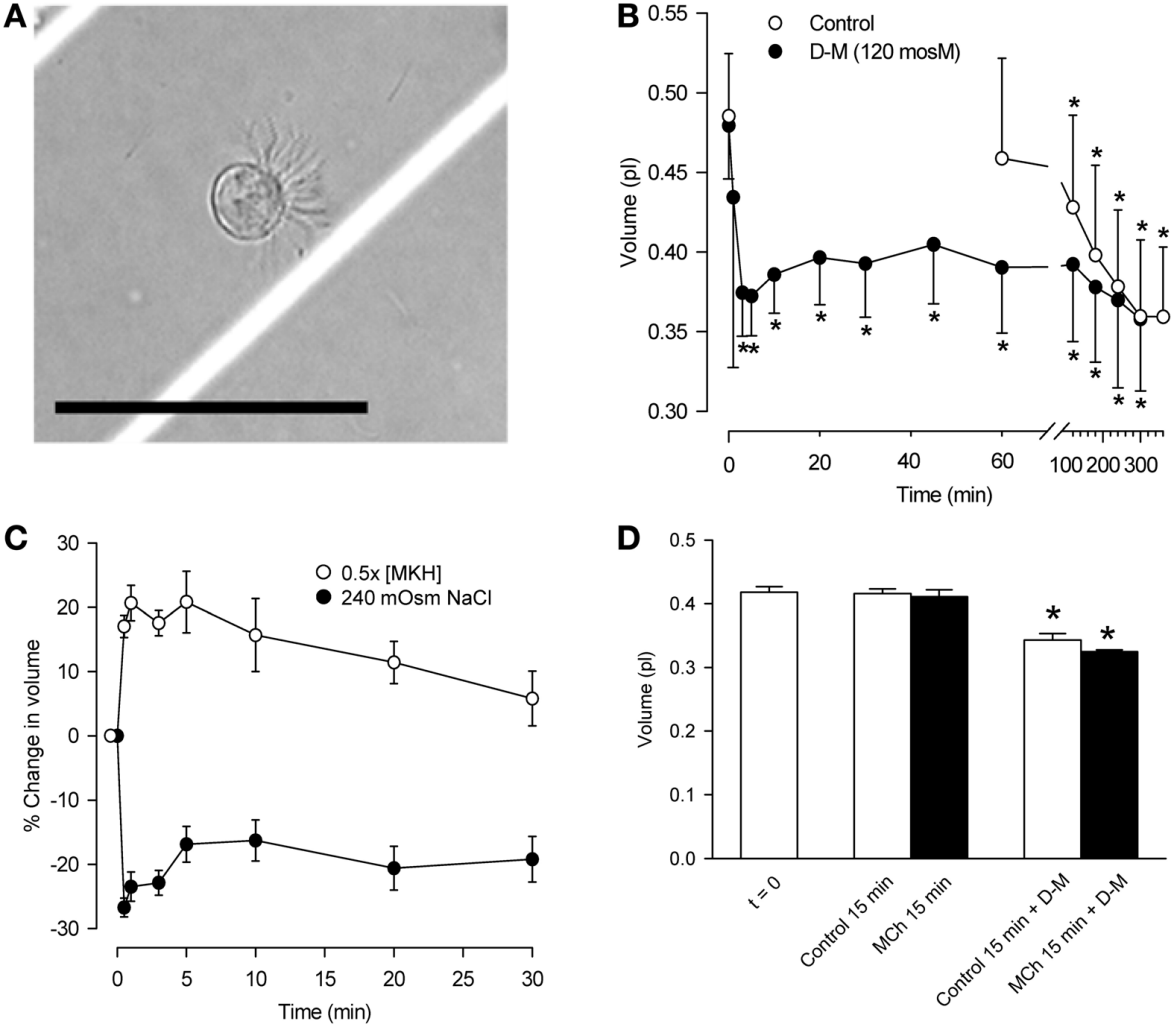
**Characterization of dispersed tracheal epithelial cells. (A)** Typical single, ciliated epithelial cell showing rounded appearance and polarized clustering of cilia. Bar = 50 μm. **(B)** Time-course of epithelial cell volume in un-stimulated and D-M (120 mOsM)-challenged cells after the 1 h equilibration period in MKH solution. *n* = 4. ^*^Significantly different compared to *t* = 0 min. **(C)** Cell volume responses of epithelial cells following challenge with half-strength (hypotonic) MKH solution [0.5 (MKH); *n* = 5] and hyperosmolarity achieved with NaCl (240 mOsM; *n* = 4) added to the MKH solution. **(D)** Lack of effect of MCh on cell volume decrease initiated by challenge of epithelial cells with D-M (120 mOsM). *n* = 4. ^*^Significantly different compared to *t* = 0 min.

Guinea-pigs anesthetized with sodium pentobarbital (65 mg/kg, i.p.) were sacrificed by thoracotomy and bleeding and 4.2 cm long tracheal segments were removed. After cleaning in modified Krebs-Henseleit (MKH) solution (composition below) the tracheas were cut longitudinally through the smooth muscle band, and incubated with 2 ml 0.2% protease in EMEM at 37°C for 1 h. The digestion was stopped with 10% FBS/EMEM solution at 4°C. The epithelial cells were scraped off with a scalpel blade; clumps were rinsed and triturated in 10 ml of EMEM solution containing 0.1% DNase I. The digest was centrifuged (800 rpm) for 4–5 min at 10°C. Cells pooled from several animals, the number of which was determined by the particular experiment, were re-suspended in 5 ml of MKH solution, filtered (Falcon 40 mm nylon filter) and centrifuged. The cells were suspended in 1 ml of gassed MKH solution and incubated for 1 h at 37°C, to allow for re-establishment of ion gradients. Cell suspensions were divided into aliquots for the various experimental conditions. Cell integrity was assessed microscopically after adding 0.4% trypan blue solution. A typical ciliated cell in the suspension is shown in Figure 1A.

### Cell volume measurement of dispersed cells

Cell volume was calculated from diameter measured with a cell sizer (Coulter Multisizer, Beckman Coulter, Inc.; Fullerton, CA). ~12 s was required for volume measurements. Thus, volume was decreasing during the early, ~30 s time point readings. Challenge of the cells with agents being investigated for their hyperosmolar effects on cell volume involved rapid pipetting of cell suspension (5–50 μl) into 20 ml vials containing solutions (37°C) of interest, and mixing the vials with gentle inversion. Cell size readings were begun 3–5 s later.

Challenge of cells with hypoosmolar solution was accomplished by first suspending cells in 10 ml of MKH solution, followed by rapid mixing in the vial with 10 ml of added distilled water (37°C) in order to halve the osmolarity, before volume measurements were made.

To examine the effects of isosmolar solutions, the cells in MKH were allowed to settle to the bottom of a conical tube. All the MKH solution except that trapped between the cells was aspirated. Isosmolar solution (1 ml; gassed; 37°C) was added to the cells, a 20 μl sample was mixed into a vial of isosmolar solution of the same composition, and measurements were made.

To examine the effects of a transition from isosmolar solution to hyperosmolar solution (37°C; gassed), referred to as “hyperosmolar jump,” cells (20 μl) from the isosmolar suspension were placed in a vial of hyperosmolar solution, mixed, and measurements were made.

### IPT preparation

The IPT (Munakata et al., 1988; Fedan and Frazer, 1992; Jing et al., 2008a) is a novel preparation that permits agents to be applied separately to the mucosal (intraluminal or IL) or serosal (extraluminal or EL) surfaces of the trachea while monitoring contractile or relaxant responses of the airway smooth muscle from changes in diameter. It allows assessment of the role of the epithelium in integrated responses of the organ (Jing et al., 2008b) and has been used to demonstrate that both the apical and basolateral membranes of airway epithelial cells respond to hyperosmolar challenge (Fedan et al., 2004a). After sacrifice, a 4.2 cm-section of trachea was excised, cleaned in gassed MKH solution, and mounted on a perfusion holder. When mounted, indwelling cannulae became inserted into the tracheal lumen at either end. The cannulae contained side holes for measurement of pressure at the inlet (positive) and outlet (negative) ends of the trachea, and changes in tracheal diameter were detected as changes in the inlet minus outlet pressure difference (ΔP in cm H_2_O) using a differential pressure transducer while the lumen was perfused with gassed MKH solution from the separate IL bath of MKH solution (IL bath) at a rate of 34 ml/min. The device was placed into an extraluminal bath containing gassed MKH solution. Transmural pressure was set to zero. Both baths were maintained at 37°C. The preparation was allowed to equilibrate for 1 h with MKH solution changes at 15-min intervals.

### Hyperosmolar jump in IPT

The trachea was contracted with MCh [3× 10^−7^ M; ~extraluminal EC50 (Fedan and Frazer, 1992)]. At the response plateau, the IL perfusion solution was changed abruptly from MKH solution to isosmolar K_2_SO_4_ or KBr solutions. Upon establishing a stable response, the perfusing solution was abruptly changed to hyperosmolar solution (120 mOsM) of the same osmolyte.

#### Confocal imaging and transepithelial V_t_ measurement

A custom chamber (RC-50 Imaging Chamber; Warner Instruments; Hamden, CT) was used to image the height of living epithelium in tracheal segments while simultaneously measuring *V*_*t*_. A 3.5-mm section of trachea was removed as described above, cleaned in MKH solution, slit longitudinally through the smooth muscle, and mounted onto the chamber. Apical and basolateral chambers were perfused (1 ml/min) independently with gassed MKH solution (37°C). Using “T” junctions, the inflow lines for the apical and basolateral chambers, containing MKH solution, were in continuity with silver/silver chloride-agar bridge voltage electrodes containing 0.9% NaCl to measure *V*_*t*_ under open-circuit conditions with a voltage/current clamp amplifier (DVC 1000; World Precision Instruments, Inc., Sarasota, FL).

The chamber was mounted on a Zeiss LSM 510 laser confocal microscope. Confocal microscopy-palette was applied to the image stacks to indicate the intensity of the fluorescent cellular stain with color scale of red/white-yellow-green-blue representing highest to lowest intensity, respectively. Epithelial cell thickness was measured using the orthogonal view/measurement function and 3-D projections of the cell layer were constructed about the *z*-axis using Zeiss image software.

Following perfusion with MKH solution for ~60 min, the fluorescent dye, calcein (15 μ M), was added to the apical perfusate for 30 min to load the epithelial cells. After a 30-min washout with MKH solution to remove extracellular calcein, control images of the un-stimulated trachea were taken.

The remaining procedures were done in such a way as to mimic the conditions used in the IPT preparation (see above). The basolateral chamber was perfused with MKH solution containing MCh (3× 10^−7^ M); from this point onward delivery of MCh was continuous. Confocal images were taken after 15–20 min. In one series of experiments, the apical bath was perfused with MKH solution while making cumulative additions of D-M to elevate osmolarity. In a second series of experiments, isosmolar solutions of D-M or urea dissolved in distilled water were delivered to the apical bath followed by D-M or urea dissolved in distilled water to create a hyperosmolar jump.

In another series of experiments the effects of selected pharmacological cytoskeleton/microtubule-interfering blockers on epithelial cell height were investigated. These included colchicine, erythro-9-(2-hydroxy-3-nonyl)adenine (EHNA), cytochalasin B and D, nacodazole and latrunculin B. In these experiments the tracheal segments were exposed to basolateral MCh before and during hyperosmolar challenge.

### Bioelectric measurements in tracheal segments: ussing chamber

An Ussing chamber (World Precision Instruments) was used to measure changes in *V*_*t*_ and transepithelial resistance (*R*_*t*_) in response to various solutions and agents. A tracheal segment was prepared as described above, reflected open and anchored across an aperture of 0.125 cm^2^ to separate the apical and basolateral hemi-chambers. Both hemi-chambers (5 ml each) were perfused separately with recirculating, gassed MKH solution (37°C). Two silver/silver chloride-agar bridge voltage electrodes containing 0.9% NaCl, and two silver/silver chloride-agar bridge current electrodes containing 0.9% NaCl, were placed, one of each type in each hemi-chamber, to monitor *V*_*t*_ and deliver current, respectively. Isotonic NaCl-containing bridge electrodes were used instead of 3 M KCl-containing bridges to prevent possible changes in osmolarity arising from KCl diffusion from the electrodes. The preparations were allowed to equilibrate with MKH solution changed at 15–30 min intervals. *V*_*t*_ was measured under open-circuit conditions (DVC 1000 or EVC 3000; World Precision Instruments, Inc.). Square-wave pulses (5 μA, 5 s) were delivered at 50-s intervals in order to obtain *R*_*t*_ from Ohm's law.

#### Preparations of isosmolar and anisosmolar solutions

A freezing point depression osmometer (Osmette A Osmometer; Precision Systems Inc.; Natick, MA), was used to determine the osmolarity of solutions (±2 mOsM standard error). The osmometer was calibrated before use with reference solutions (100 and 500 mOsM). Isosmolar solutions were matched to the osmolarity of MKH solution prepared for each experiment.

### MKH solution

MKH solution (pH 7.4; osmolarity of 281 ± 5 mOsM; 37°C) contained 113 mM NaCl, 4.8 mM KCl, 2.5 mM CaCl_2_, 1.2 mM KH_2_PO_4_, 1.2 mM MgSO_4_, 25 mM NaHCO_3_, and 5.7 mM glucose, and was gassed with 95% O_2_/5% CO_2_. All reagents were from Sigma-Aldrich (St. Louis, MO).

### Statistical analysis

The results are expressed as means ± SE. All data were normally distributed. ANOVA for repeated measures was utilized to detect differences when multiple measurements were made using a single sample. In experiments in which two measurements were made using a single sample, and readings were taken before (control) and after an experimental manipulation, Student's *t*-test for paired samples was employed to detect significant differences. Student's *t*-test for non-paired data was employed to detect significant differences when appropriate for single comparisons made between two unpaired samples. *P* < 0.05 was considered significant. *n* is the number of separate experiments. *n*-values for perfused trachea and confocal experiments represent results obtained using tracheas from separate animals; *n*-values for dispersed cells represent separate experiments in which cells pooled from several tracheas were employed.

## Results

### Initial characterization of dispersed epithelial cells

The cell suspension consisted primarily of single cells or doublets of ciliated and non-ciliated cells (Figure 1A). In ciliated cells, cilia were clustered and beating was evident. The volume of un-stimulated cells was ~0.42–0.48 pl. The cells excluded trypan blue (85–93%) for at least 5 h, during which cilia continued to beat.

The volume of control cells did not change over a 1-h period (Figure 1B) but decreased significantly after 120 min, reaching a value of ~0.36 pl after 5 h. In response to 120 mOsM D-M or 240 mOsM NaCl, volume decreased rapidly, reaching a maximum by ~30 s to 1 min. Volume then increased somewhat at ~10–20 min, reflecting modest regulatory volume increase (RVI), and remained constant over 60 min. By 120 min cell volume declined similarly to the control cells. Based on these results, experiments with dispersed cells lasted no longer than 30 min.

Responses to hypoosmolar challenge (Figure 1C) were examined. After exposing cells to half-osmolar MKH an immediate swelling, maximal by 1 min, was followed by regulatory volume decrease (RVD) over a 30-min period. To examine relaxant effects following exposure of epithelium to isosmolar and hyperosmolar solutions in previous studies, the IPT preparations were first contracted with EL MCh (3× 10^−7^ M; see Figure 4). Cell volume was unaffected by MCh (3× 10^−7^ M) during a 15-min incubation (*n* = 7; not shown). MCh had no effect on D-M-induced cell shrinkage responses (Figure 1D); therefore, MCh was omitted in many of the remaining experiments.

### Osmolar concentration dependence of cell shrinkage in dispersed cells

**Figure 2:**
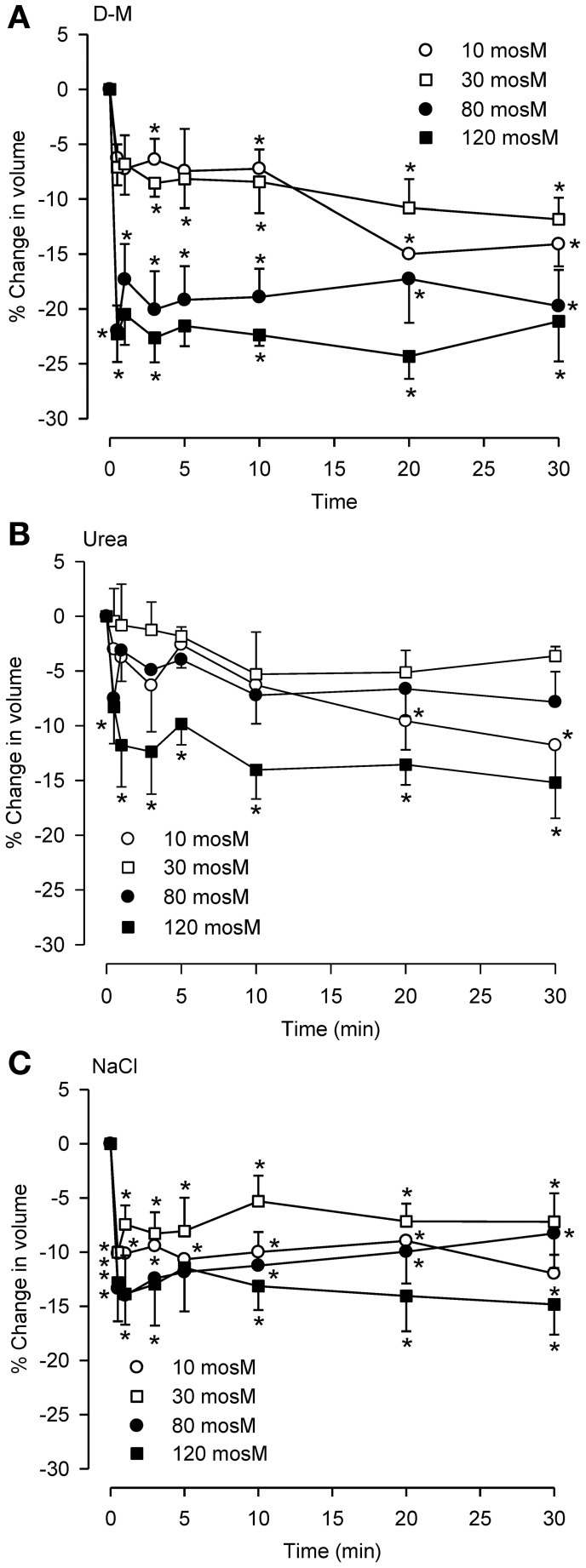
**Osmolar concentration-dependence of the effects of D-M (A), urea (B), or NaCl (C) on volume of dispersed epithelial cells. (A–C), *n* = 4, 5–9, and 5–9, respectively.** ^*^Significantly different compared to *t* = 0 min.

To compare reactivity of dispersed epithelial cells to hyperosmolar challenge with those observed previously in the IPT, we investigated the osmolar time- and concentration-dependencies of cell shrinkage using D-M (a nonionic, impermeant osmolyte), urea (a nonionic, permeant osmolyte) and NaCl (an ionic osmolyte; Figure 2). For D-M and NaCl, significant cell shrinkage was stimulated at 10 mOsM and was concentration-dependent up to 120 mOsM. Urea showed comparable reactivity. Urea and NaCl caused ~15% shrinkage, whereas D-M caused ~25% shrinkage, at the highest osmolyte concentrations. There was little evidence of RVI in these experiments, except at 80 and 120 mOsM NaCl. At 30 mOsM D-M and urea, a concentration reported earlier that approximates the EC50 of the osmolytes for relaxation of the IPT, the reduction in volume was less than half of the maximal amount of shrinkage.

### Isosmolar solution effects in dispersed epithelial cells

Previously (Fedan et al., 2004a), we reasoned that if EpDRF release in response to hyperosmolar challenge was triggered by cell shrinkage *per se* that a relaxation response could also be triggered by shrinkage under isosmolar cell shrinkage conditions. Therefore, whether isosmolar challenge of dispersed cells elicits shrinkage was examined.

**Figure 3:**
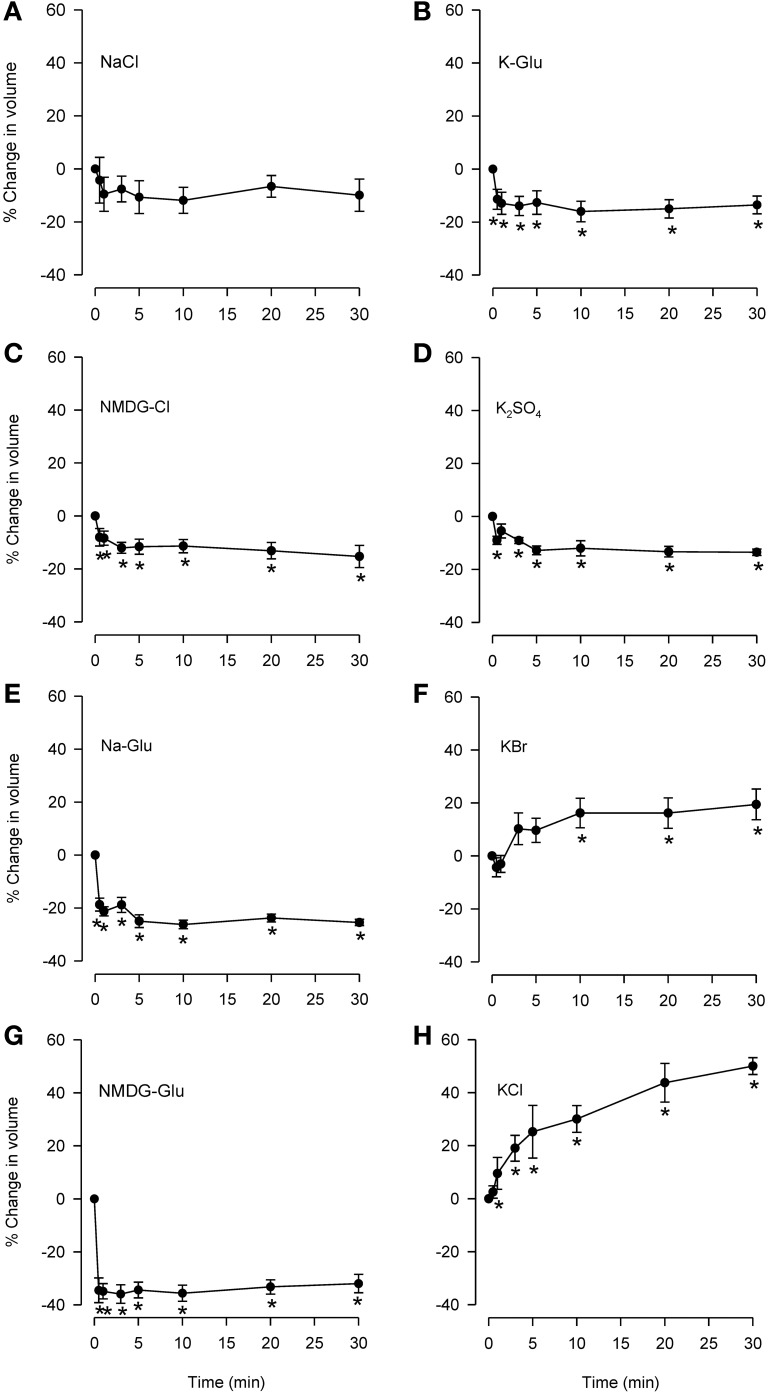
**Effects of isosmolar solutions of ionic permeant and impermeant osmolytes on volume of dispersed epithelial cells.** Cell volume was measured after the cells were placed into isosmolar solutions containing NaCl **(A; *n* = 6)**, NMDG-Cl **(C; *n* = 6)**, Na-Glu **(E; *n* = 6)**, NMDG-Glu **(G; *n* = 6)**, K-Glu **(B; *n* = 6)**, K_2_SO_4_ **(D; *n* = 6)**, KBr **(F; *n* = 6)**, and KCl **(H; *n* = 6)**. The order of increasing effectiveness at causing volume change was: K_2_SO_4_ = KGlu = NMDG-Cl (~15% decrease) < Na-Glu < NMDG-Glu (35% decrease). KCl and KBr caused increases in cell volume (~50 and 15%, respectively). D-M and urea could not be studied using this method. ^*^Significantly different compared to *t* = 0 min.

Isosmolar NaCl did not significantly affect cell volume (Figure 3A), although a small decrease was seen consistently. Isosmolar NMDG-Cl (Figure 3C) containing the impermeant cation produced a comparable cell shrinkage that was significant. Replacement of Cl^−^ with the impermeant anion Glu in Na-Glu (Figure 3E) caused a greater shrinkage response than NaCl or NMDG-Cl. Replacement of Na with NMDG along with substitution of Cl^−^ with Glu (Figure 3G) resulted in a large shrinkage response (~35%). (D-M and urea could not be examined because cell sizing is dependent on solute conductivity).

Isosmolar KCl (Figure 3H) initiated a large cell swelling response, but in the IPT did not cause relaxation (Fedan et al., 2004a). The swelling effect could have resulted from accumulation of intracellular Cl^−^ from a solution containing 144 mM Cl^−^ (greater than 122.8 mM in MKH solution) in a less negative cytoplasm resulting from depolarization of the membrane by 144 mM K^+^. We explored this notion using K^+^ salts with Cl^−^ substitutions. Isosmolar KBr (Figure 3F) initiated swelling that was approximately half that produced by isosmolar KCl; RVD was not evident. In contrast to KCl and KBr, both K-Glu and K_2_SO_4_ caused shrinkage without RVI (Figures 3B,D).

No RVI or RVD was observed during responses to these osmolytes under isosmolar conditions.

### Hyperosmolar challenge of IPT following perfusion with isosmolar solution (hyperosmolar jump protocol)

**Figure 4:**
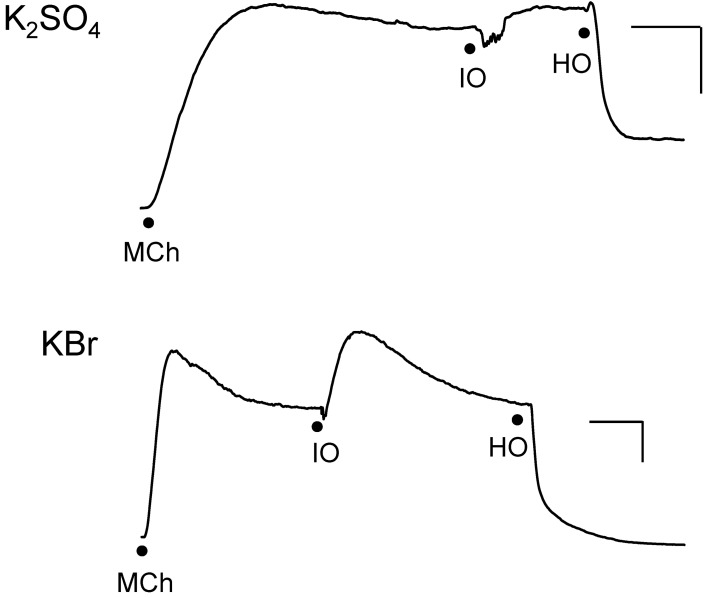
**Responses of IPT to perfusion with isosmolar (IO) K_2_SO_4_ (Top) or KBr (Bottom), followed by hyperosmolar (HO; 120 mOsM) challenge with the same osmolyte (hyperosmolar jump).** These results are representative of *n* = 4 experiments for K_2_SO_4_ and *n* = 6 for KBr, in which contraction (shown) to isosmolar K_2_SO_4_ or KBr or no effect were observed (not shown). The discontinuities in the responses after the isosmolar additions occurred during perfusion solution changeover. Vertical bar, 5 cm H_2_O; horizontal bar, 5 min.

We investigated the effects of isosmolar KBr and K_2_SO_4_ in the IPT inasmuch as these two osmolytes affected cell volume oppositely (see Figure 3). Both agents initiated contractions (Figure 4) or had no effect (not shown) when applied to the IL bath, in the manner seen earlier for KCl. K^+^, which is present in high concentration (144 mM) in these isosmolar solutions (compared to MKH solution), would be expected to diffuse across the epithelium in sufficient quantities to cause depolarization of the smooth muscle and contraction. But adding 120 mOsM of KBr and K_2_SO_4_ triggered large and long-lasting relaxations.

### Hyperosmolar challenge of dispersed epithelial cells following incubation in isosmolar solution (hyperosmolar jump protocol)

**Figure 5:**
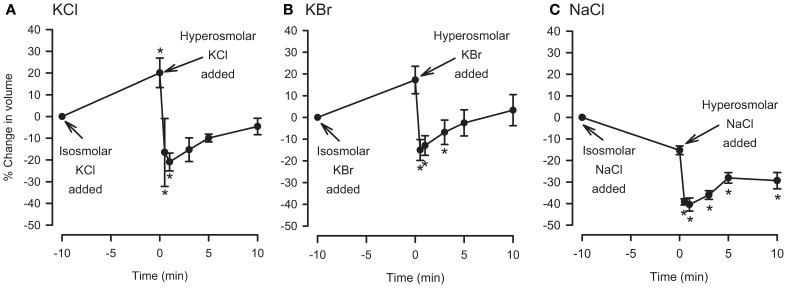
**Effects of isosmolar osmolyte solutions, and hyperosmolar osmolyte solutions added in the presence of isosmolar solution (osmolar jump), on volume of dispersed epithelial cells.** *n* = 5, 5, and 4 for KCl, KBr, and NaCl, respectively. ^*^Significantly different compared to *t* = 0 min.

For comparison, the hyperosmolar jump protocol used in the IPT was applied to dispersed cells. The cells were first incubated with isosmolar KCl, KBr, or NaCl for 10 min, after which they were challenged with added 120 mOsM of KCl, KBr, or NaCl. KCl and KBr were chosen for these experiments because under isosmolar conditions they had caused cell swelling but not relaxation of the trachea, and NaCl was chosen because it also did not cause consistently cause relaxation under isosmolar conditions (Fedan et al., 2004a). As expected, placement of cells in isosmolar KCl or KBr initiated cell swelling, and isosmolar NaCl led to cell shrinkage which was reproducible but not significant (Figure 5). After addition of KCl, KBr, or NaCl to elevate osmolarity, the cells immediately shrank to levels that were both less than the *t* = 0 min and *t* = −10 min values. RVI was swift; volume was gained by *t* = 5 min and returned to the *t* = −10 min values in the cases of KCl and KBr. Hyperosmolar NaCl-challenged cells lost 40% of their volume and did not volume regulate to the *t* = −10 min values. In contrast, hyperosmolar solution addition to tracheas perfused with isosmolar or MKH solution stimulated a relaxation that remained at a stable plateau (Figure 4).

### Effects of Na^+^ and Cl^−^ channel inhibitors on dispersed cells and their responses to MCh and hyperosmolar solutions

**Figure 6:**
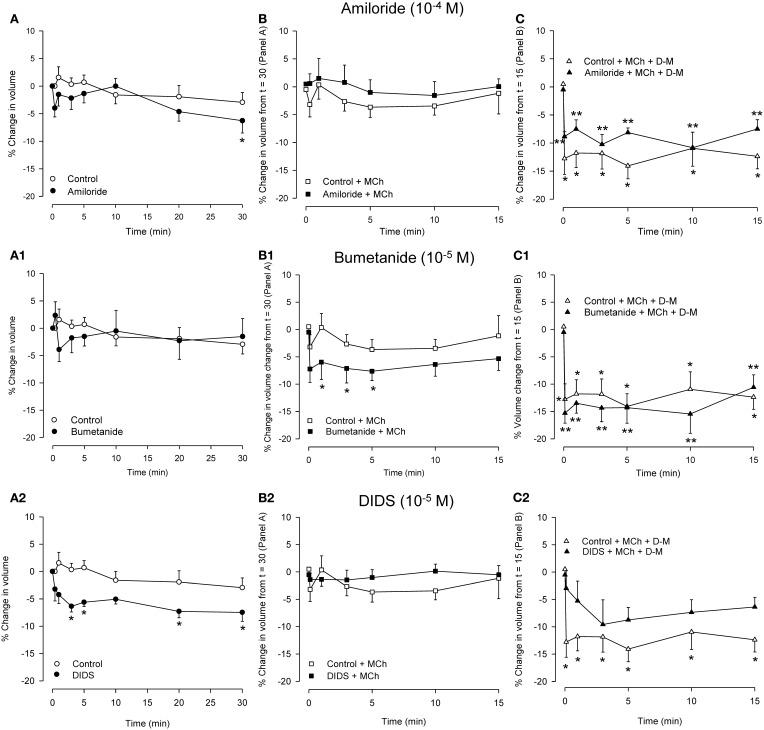
**Effects on cell volume of transport blockers alone (A panels), and the effects of the blockers on volume responses to MCh (B panels; 3 × 10^−7^ M) and D-M in the presence of the blockers and MCh (C panels; 30 mOsM) in dispersed cells.** Cells were incubated with the blocker for 30 min (**A** panels), MCh plus blocker for 15 min (**B** panels), or MCh plus blocker plus D-M for 15 min (**C** panels). Control cells were not incubated with blockers. **(A)** ^*^Significantly different compared to Control at *t* = 0 min. **(B)** ^*^Significantly different from Control + bumetanide at *t* = 15 min. **(C)** ^*^Significantly different from Control + MCh at *t* = 15 in; ^**^significantly different from blocker + MCh at *t* = 15 min. *n* = 4 – 7.

Because relaxation of the MCh-contracted preparations in response to hyperosmolar challenge was inhibited by amiloride, DIDS and NPPB but not by bumetanide (see Introduction), experiments were conducted using dispersed cells to examine the effects of these blockers on responses to MCh and hyperosmolar solutions. Osmolarity was raised using D-M, rather than NaCl, to avoid changes in ion gradients. The IPT protocol was mimicked: cells were incubated with an inhibitor for 30 min, MCh (3× 10^−7^ M) was applied, and 15 min later D-M was added to elevate osmolarity while MCh remained. Cells from separate preparations were used to obtain control data and to evaluate the effects of the ion transport blockers, MCh in the presence of ion transport blockers, and D-M in the presence of the ion transport blockers and MCh. The results are depicted in Figure 6.

In control cells volume was stable over 30 min. Amiloride (10^−4^ M) reduced cell volume after 30 min. MCh (3× 10^−7^ M), either in the absence or presence of amiloride, had no effect. D-M (30 mOsM) evoked significant shrinkage both in the absence or presence of amiloride. Compared to the control cells, the shrinkage was attenuated at most time points when amiloride was present.

Bumetanide (10^−5^ M) had no effect on cell volume, but, in the presence of bumetanide, MCh induced significant volume decrease at early time points. In cultured guinea-pig tracheal epithelial cells (Fedan et al., 2007) basolaterally-applied MCh stimulates Cl^−^ efflux. Coupled with inhibition by bumetanide of Cl^−^ influx *via* Na^+^,K^+^,2Cl^−^-cotransport, it is possible that MCh-stimulated Cl^−^ efflux resulted in a decrease in intracellular Cl^−^ level that promoted shrinkage. Bumetanide had negligible effects on shrinkage responses to D-M, and, if anything, had a small potentiating effect.

Cell volume was decreased in the presence of DIDS (10^−4^ M); NPPB (10^−5^ M) did not have this effect (not shown). It is difficult to explain the cell shrinkage in the context of inhibited Cl^−^ efflux, which would evoke cell swelling, and the fact that DIDS and NPPB differed in their effects. Neither blocker affected volume in the presence of MCh (NPPB not shown; *n* = 5), and both agents inhibited D-M-induced cell volume reduction, DIDS to a greater degree (NPPB not shown; *n* = 5).

### Lack of effect of calcein on epithelial ion transport

Before the fluorescent intracellar cellular dye, calcein, was used in bioelectric and confocal microscopy experiments described below, we first investigated whether it had any effects on ion transport. Both calcein (1.5 × 10^−5^ M) dissolved in DMSO (*n* = 4) and DMSO alone (*n* = 4) caused small hyperpolarizations (~1 mV; *P* > 0.05). After 30 min of incubation, MCh (3 × 10^−7^ M) was applied to the serosal bath; the resulting ~1 mV hyperpolarization was not affected by calcein (*P* > 0.05). After 15 min, 120 mOsM D-M applied to the apical bath caused depolarization; subsequently-added 240 mOsM D-M elicited a further depolarization. There were no differences in the two responses to D-M in the absence and presence of calcein (*P* > 0.05). Calcein, DMSO and MCh had no effects on *R*_*t*_. D-M increased *R*_*t*_ in a concentration-dependent manner, but calcein had no effect on these responses (*P* > 0.05). It was concluded that calcein would not affect *V*_*t*_ responses in confocal microscopy experiments.

### Effects of hyperosmolar and isosmolar solutions on *in situ*, adherent epithelium: bioelectric responses and confocal microscopy

**Figure 7:**
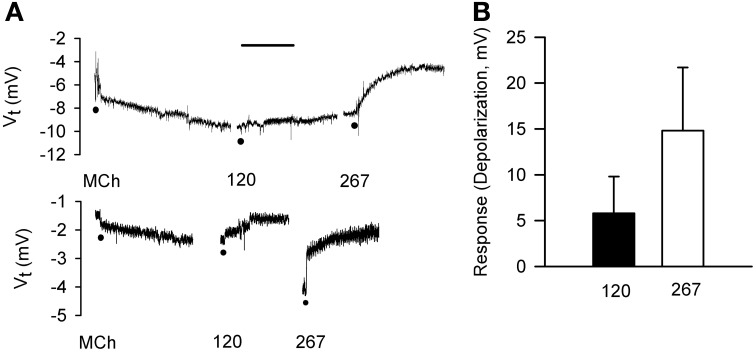
**Bioelectric responses of tracheal segments to MCh (basolateral; 3 × 10^−7^ M) and D-M (apical; 120 and 267 mOsM) obtained in the confocal imaging chamber. (A)** Representative responses to MCh and D-M. Basal *V*_*t*_ average from all experiments was 6.7 ± 1.4 mV (*n* = 6). Bar = 5 min. **(B)** Summary of concentration-dependence of D-M-induced depolarization. Bioelectric responses to D-M in concentrations less than 120 mOsM were rarely produced. *n* = 2 and 2 for 120 and 267 mOsM D-M.

For comparison to dispersed cells, cell volume changes of adherent epithelial cells were measured in relation to electrophysiological changes using protocols employed in IPT experiments. The goal of comparing the time-courses of bioelectric and volume responses in “real time” proved to be infeasible, as the 2–3 min required for processing images was too long to permit moment-to-moment comparisons with bioelectric changes. First, we validated the preparation. Serosally-applied MCh (3× 10^−7^ M) elicited hyperpolarization (5.6 ± 2.5 mV; *n* = 6). The subsequent addition of 120 mOsM and 267 mOsM D-M to the mucosal chamber evoked concentration-dependent depolarization responses (Figure 7). Occasionally 120 mOsM D-M triggered hyperpolarization. These results are consistent with our previous findings from IPT and Ussing preparations.

**Figure 8:**
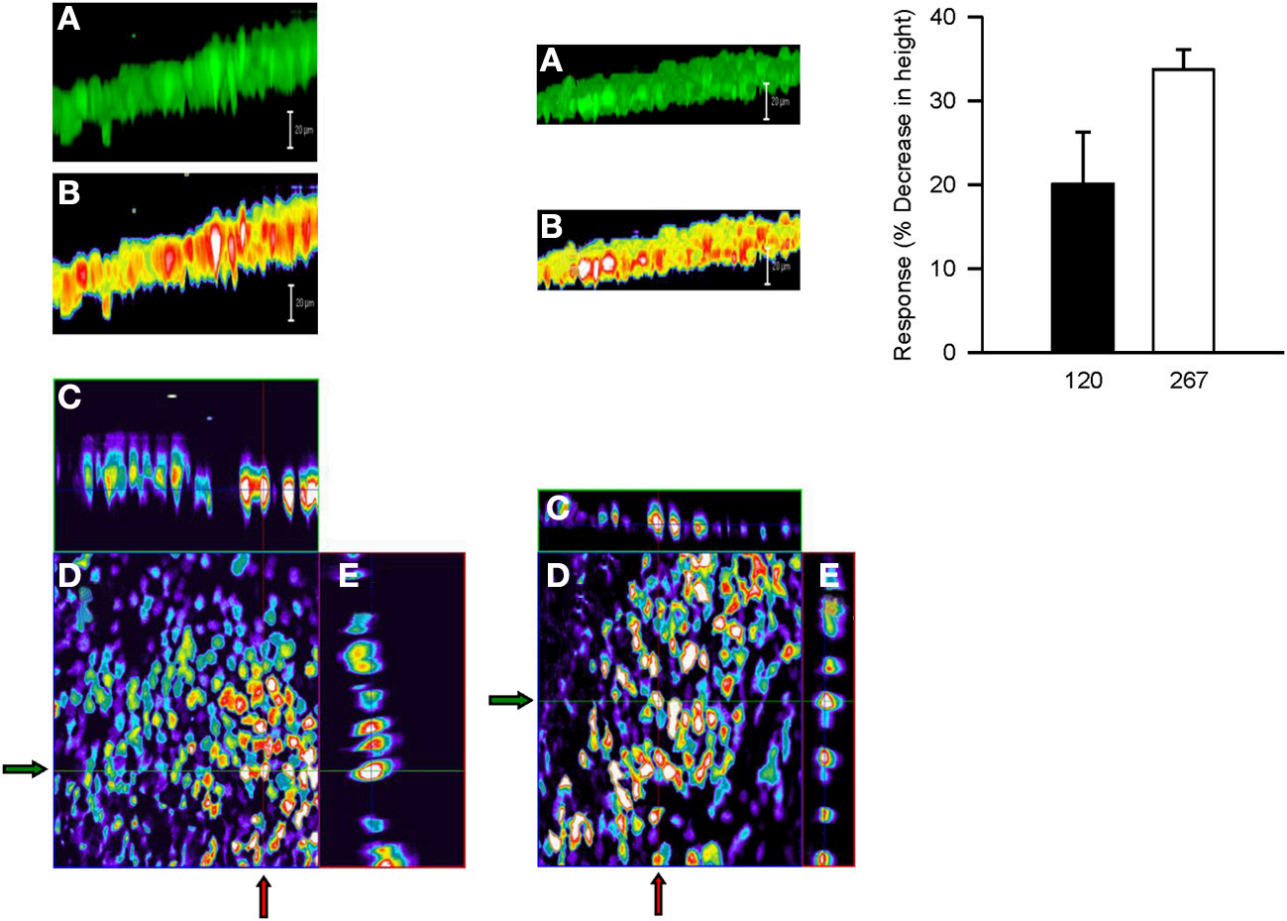
**Representative confocal micrographs showing cell height of calcein-loaded control epithelium (left column) and after apical challenge with 120 mOsM D-M (middle column). (A)** Three dimensional reconstruction of the image stack on the *z*-axis. Bar = 20 μM. **(B)** panel **(A)** in pseudocolor. Bar = 20 μM. **(C)** x-z plane (vertical red lines at arrows). **(D)** x-y plane of a single section in the middle of the image stack. **(E)** y-z plane (horizontal green lines at arrows). The bar graph in the right column depicts the concentration-dependence of volume responses to apically-applied D-M. Cell height was quantified from the image stack on the *z*-axis in confocal images and normalized with respect to control values. 120 mOsM D-M, *n* = 5; 267 mOsM D-M, *n* = 3.

Serosal MCh (3 × 10^−7^ M) addition had no effect (5.8 ± 5.6%) on cell height (*P* > 0.05), nor did mucosal D-M in concentrations less than 120 mOsM (not shown). This is in contrast to the finding that 10 mOsM D-M caused shrinkage of dispersed cells (above) and EpDRF release and ion transport alterations (previous studies). Nevertheless, after 15–20 min of exposure, 120 and 267 mOsM D-M caused concentration-dependent shrinkage (Figure 8), up to ~35% at 267 mOsM, over the same range it caused depolarization (Figure 7).

**Figure 9:**
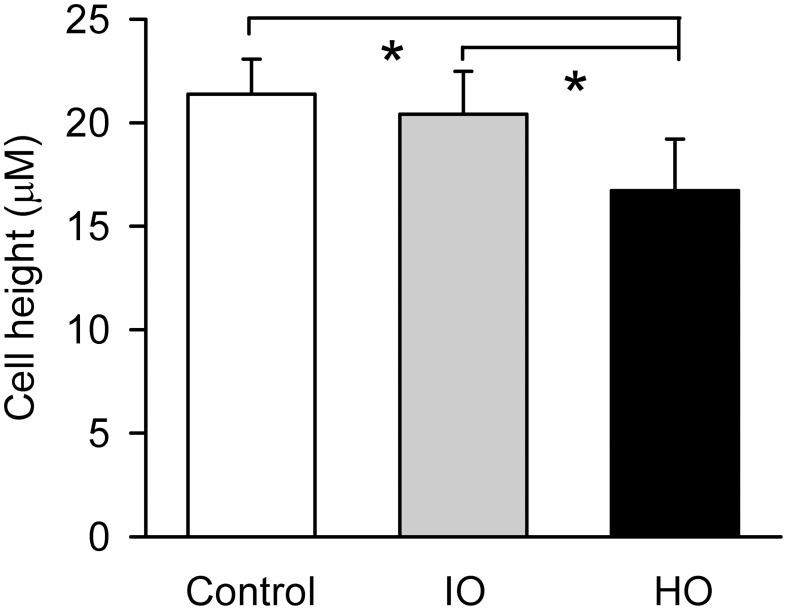
**Effects of apical isosmolar (IO) and hyperosmolar (HO) D-M on epithelial cell height in the confocal chamber.** ^*^Significantly less than Control and IO. *n* = 4.

The effects of isosmolar D-M and urea on cell volume could be investigated in the confocal apparatus. Neither osmolyte affected cell height (Figure 9; compare to Figure 3). Upon addition of 120 mOsM D-M or urea to the isosmolar solutions, D-M caused a decrease in cell height but urea was without effect (not shown; *n* = 4). Hyperosmolar urea, in contrast, was equiactive with D-M and other osmolytes in relaxing the trachea (Fedan et al., 2004a).

### Effects of cytoskeleton/microtubule-interfering inhibitors on epithelial bioelectric responses: ussing chamber

**Figure 10:**
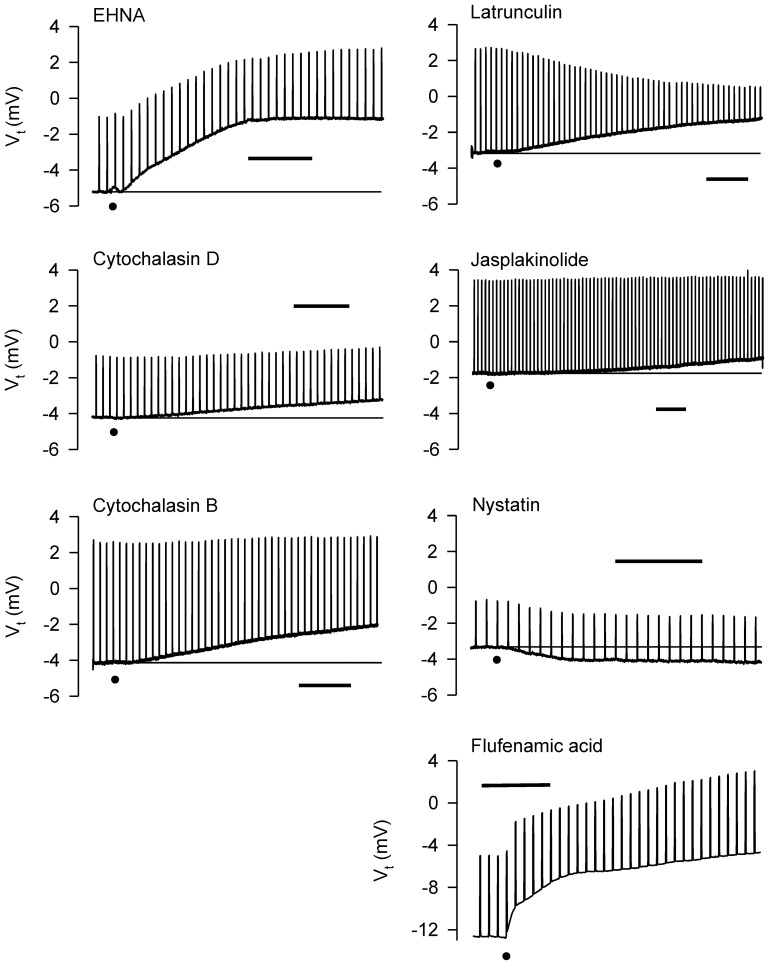
**Representative effects of apically-applied cytoskeleton/microtubule-interfering agents colchicine (2 × 10^−4^; *n* = 4), EHNA (5 × 10^−4^ M; *n* = 4), cytochalasins B (5 × 10^−7^ M; *n* = 6), and D (5 × 10^−7^ M; *n* = 6), latrunculin B (5 × 10^−6^ M; *n* = 4), jasplakinolide (5 × 10^−6^ M; *n* = 4), the pore-forming agent, nystatin (2.6 × 10^−4^ M; *n* = 6), and the HICC inhibitor, flufenamic acid (10^−4^ M), on *V*_*t*_ and *R*_*t*_ of tracheal segments in Ussing chambers.** DMSO (0.1%), the solvent for these agents (except colchicine), was without effect on *V*_*t*_ and *R*_*t*_. A summary of the results of all experiments is shown in Figure 11. Bar = 400 s. The baselines are provided in some cases for reference. The dots indicate the additions of the agents.

**Figure 11:**
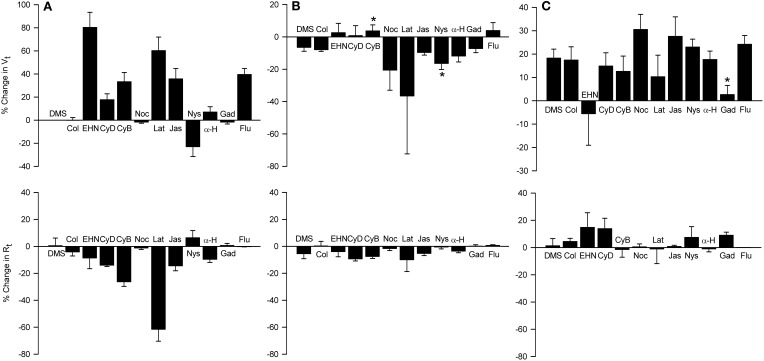
**Effects of apically-applied cytoskeleton/microtubule- interfering agents, pore-forming agents and hypertonicity-induced cation channel blockers, on *V*_*t*_ and *R*_*t*_ (A), *V*_*t*_ and *R*_*t*_ responses to MCh (B), and *V*_*t*_ and *R*_*t*_ responses to 120 mOsM D-M (C).** Top row: % change in *V*_*t*_ values above zero signify depolarization; negative values signify hyperpolarization. Bottom row: % change in *R*_*t*_ values above zero signify an increase in; negative values signify a decrease in *R*_*t*_. DMSO (DMS, 0.1%) was the solvent for all agents except colchicine and gadolinium, which were dissolved in saline. Agent abbreviations and their concentrations are: Col, colchicine (2 × 10^−4^ M; *n* = 4); EHN, EHNA (5 × 10^−4^ M; *n* = 4); CyD and CyB, cytochalasins D and B, respectively (2 × 10^−5^ M; *n* = 6 and 6); Noc, nocodazole (2 × 10^−5^ M; *n* = 4); Lat, latrunculin B (5 × 10^−6^ M; *n* = 4); Jas, jasplakinalide (5 × 10^−6^ M; *n* = 4); Nys, nystatin (2.6 × 10^−4^ M; *n* = 6); α-H, α-hemolysin (100 units/ml; *n* = 4); Gad, gadolinium (10^−4^ M; *n* = 4); Flu, flufenamic acid (10^−4^ M; *n* = 5). *A*, summary of the responses elicited by the agents; *B*, responses to MCh obtained in the presence of the agents; *C*, responses to D-M in the presence of the agent and MCh (3 × 10^−7^ M). ^*^Significantly different from DMSO (*n* = 4).

Cytoskeletal re-arrangements accompany volume change in cells. EHNA, colchicine, nocodazole, cytochalasins B and D, and latrunculin B did not inhibit relaxation responses of the IPT to D-M, whereas latrunculin B potentiated the responses (Fedan et al., 2004b). Little is known of the effects of these agents on airway epithelial ion transport. Therefore, we investigated their effects on *V*_*t*_ and *R*_*t*_ and bioelectric responses to MCh and D-M and cell volume responses (Figures 10, 11).

The dynein inhibitor, EHNA (0.5 mM), the actin microfilament inhibitors, cytochalasins B and D (both at 5 × 10^−7^ M) and latrunculin B (5 × 10^−6^ M), and jasplakinolide (5 × 10^−6^ M; stabilizes actin filaments) evoked depolarization; the response to EHNA was robust. The decrease in *V*_*t*_ in response to EHNA not accompanied by a change in *R*_*t*_ suggests a decrease in transcellular ion transport. The remaining agents variously decreased *R*_*t*_. The decreases in *R*_*t*_ caused by the cytochalasins and latrunculin B may explain the depolarization responses they initiated. The depolarization caused by jasplakinolide (~35%) was greater than the change in *R*_*t*_ (~15%), suggesting that transcellular ion transport was inhibited. Colchicine (0.2 mM) and nocodazole (2.5 × 10^−5^ M) (both inhibit microtubule polymerization) had no effect on *V*_*t*_ and *R*_*t*_. These findings indicate that the cytoskeleton and microfilaments regulate ion transport variously *via* transcellular and paracellular pathways.

MCh (3× 10^−7^ M; Figure 11) elicited hyperpolarization without affecting *R*_*t*_; this effect was not due to DMSO vehicle [see also Figure 7 and Johnston et al. (2004)]. EHNA and the cytochalasins appeared to inhibit the response to MCh, but only cytochalasin B produced a significant inhibitory effect. This observation agrees with the finding that cytochalasin B inhibited contractions of IPT to MCh (Fedan et al., 2004b). The remaining agents did not affect responses to MCh.

Only latrunculin B potentiated 120 mOsM D-M-induced relaxation of MCh-contracted IPT (Fedan et al., 2004b). Therefore, we investigated the effects of these blockers on *V*_*t*_ and *R*_*t*_ responses to D-M. In the presence of MCh, none of the agents affected depolarization or *R*_*t*_ responses to D-M (Figure 11). Collectively these findings indicate that the bioelectric response of airway epithelium to D-M is not regulated by the cytoskeleton.

### Effects of cytoskeleton/microtubule-interfering inhibitors on dispersed epithelial cell volume responses

**Figure 12:**
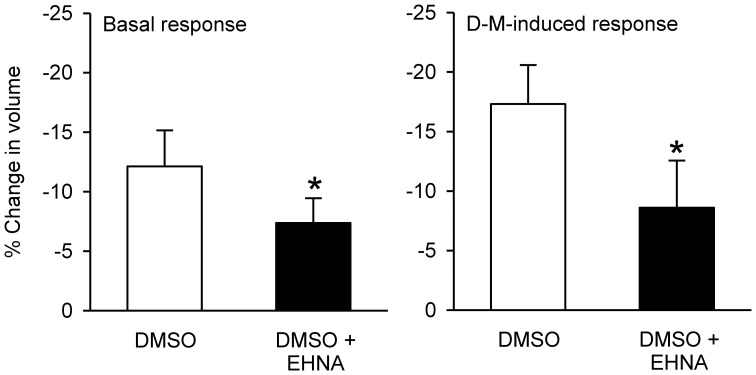
**Effects EHNA (5 × 10^−4^ M) on cell volume and volume responses to D-M. Left panel** (Basal response): % reduction in volume under basal conditions after addition of DMSO or EHNA dissolved in DMSO, during the 30 min incubation period. **Right panel** (D-M induced response): % reduction in volume after subsequent addition of 120 mOsM D-M dissolved in DMSO. In these experiments a control volume measurement was made before incubating the cells with vehicle or agents; the cells used in each replicate were from the same preparation. ^*^Significantly different from D-M. Basal response, *n* = 4; DMSO-induced response.

In preliminary experiments, DMSO, the solvent for most of these agents, reduced cell volume even at the lowest concentration (0.1%) needed to dissolve the inhibitors. Therefore, a DMSO control was included in every experiment. EHNA dissolved in DMSO produced less cell shrinkage than DMSO itself (Figure 12); the other agents had no effect compared to control (DMSO or vehicle control; not shown). After incubation with DMSO or agent dissolved in DMSO, 120 mOsM D-M in DMSO vehicle-containing MKH solution was added and cell volume was measured. EHNA inhibited responses to D-M (Figure 12). In these experiments colchicine, cytochalasins B (*n* = 6) and D (*n* = 6), nocodazole (*n* = 4) and latrunculin B (*n* = 4) had no effect (not shown). These findings agree with the lack of effect of these inhibitors on cell volume responses to hyperosmolar challenge in other cells (Foskett and Spring, 1985; Hallows et al., 1991, 1996).

### Effects of nystatin and α-hemolysin on epithelial bioelectric responses: ussing chamber

If alterations in ion transport induced by hyperosmolar solutions are required for EpDRF release, then disrupting ion transport with nystatin [increases membrane cation permeability (Akaike and Harata, 1994)] and α-hemolysin [a pore-forming protein (Panchal et al., 2002)] would inhibit relaxation. However, IPT responses to D-M were potentiated, not inhibited, by apical nystatin and apical α-hemolysin had no effect (Fedan et al., 2004b). Nystatin (2 × 10^−4^ M; apical) evoked hyperpolarization (Figure 11). α-Hemolysin (100 U/ml M; apical) was essentially without effect. Neither agent affected *R*_*t*_. Nystatin but not α-hemolysin potentiated hyperpolarization responses to MCh (Figure 11). Neither agent affected depolarization in response to D-M (Figure 11).

### Effects of hypertonicity-induced cation channel (HICC) inhibition on epithelial bioelectric and mechanical responses

Inhibition of relaxation of the IPT to hyperosmolar challenge by amiloride suggests that EpDRF release is linked to epithelial Na^+^ channels. Two of the three HICCs are sensitive to amiloride (Hoffmann et al., 2009; Numata et al., 2012) and could have been affected by amiloride. To evaluate this possibility the effects of the HICC blockers, gadolinium and flufenamic acid, were investigated in Ussing chambers (Figure 11). The effects of the two agents were different. Apically-applied gadolinium (10^−4^ M) had no effect on *V*_*t*_ and did not affect MCh-induced hyperpolarization. It did, however, inhibit D-M-induced depolarization without affecting *R*_*t*_. However, flufenamic acid (10^−4^ M) elicited a strong depolarization but was without effect on MCh- and D-M-induced responses; *R*_*t*_ also was not affected.

In separate IPT experiments, mucosal flufenamic acid did not evoke a response (in cm H_2_O; DMSO control: −0.2 ± 0.1; flufenamic acid: 0.0 ± 0.1; *n* = 5; *P* > 0.05), nor were relaxant responses to mucosally-applied 120 mOsM D-M affected (% relaxation of the MCh-induced contraction: DMSO control: 84.1 ± 16.1%; flufenamic acid: 67.8 ± 10.9%; *P* > 0.05). These findings suggest that while bioelectric responses to hyperosmolarity may involve HICCs, relaxant responses mediated by EpDRF do not.

## Discussion

Small elevations in osmolarity, comparable to those that activate forebrain osmosensory neurons (Bourque, 2008; Ciura et al., 2011), are detected by the airway epithelium and alter ion transport and elicit EpDRF release and airway smooth muscle relaxation. Earlier functional studies using the IPT indicated indirectly that the stimulus to EpDRF release after hyperosmolar challenge of epithelium results not from cell shrinkage, but from the incremental increase in osmolarity. The focus of the present study was to employ parallel strategies and protocols used in earlier functional studies in order to investigate whether EpDRF release is linked to cell shrinkage. A new characterization of some cell volume regulation properties of the epithelium also was obtained. This information will be of use for understanding the consequences of elevations in ASL during exercise and in response to therapies developed to raise the osmolarity of the ASL with osmolar agents, such as saline and D-M.

The main conclusion of this investigation is EpDRF release from adherent epithelial cells in response to hyperosmolar challenge is unrelated to volume changes in the cells. Another conclusion is dispersed epithelial cells share many volume regulation properties reported in other cell types, but their sensitivity to the volume effects of hyperosmolar challenge is substantially greater than that of adherent epithelial cells. Finally, the reactivity of dispersed cells to hyperosmolar challenge is paradoxically comparable to that of adherent cells in relation to EpDRF release but not volume change in adherent cells. The relevance of these novel findings to regulation of submucosal blood flow by apical osmolarity warrants further investigation.

The volume responses of epithelium upon hyper- and hypoosmolar challenge with ionic and non-ionic osmolytes were similar to those reported in other cells (Foskett and Spring, 1985; Hallows et al., 1991; Nielsen et al., 2007; Hua et al., 2010; Numata et al., 2012 and references in Introduction). During hyperosmolar exposures of dispersed cells RVI was evident in some but not most preparations and it was not as vigorous as that observed in other cell types [(Hallows et al., 1991, 1996; Pedersen et al., 1998; Nielsen et al., 2007; Numata et al., 2012) and reviews above]. Willumsen et al. (1994) did not find evidence of RVI in cultured human airway cells during hyperosmolar challenge. However, RVI was evident and consistent in hyperosmolar jump experiments performed on dispersed cells. Exposure of dispersed cells to halved osmolarity led to cell swelling accompanied by RVD, as observed in other cell types (Hallows et al., 1991; Pedersen et al., 1998; Nielsen et al., 2007), including Calu-3 cells (Harron et al., 2009).

Swelling of dispersed cells occurred in response to hypoosmolar challenge and during incubation with isosmolar KCl and KBr. That stimulated by KCl was observed to be halide-dependent. The anion permeability sequence for the cells (Cl > Br > SO_4_ > Glu) is somewhat distant from the permeability sequence of volume-activated Cl^−^ channels of parotid gland and HL-60 cells [Br > Cl > Glu (Arreola et al., 1995a,b)] but comparable to swelling-induced currents in Ehrlich ascites cells (Cl > Glu; Pedersen et al., 1998).

MCh had no effect on cell volume. Except for their role in long-term cell regulation (Vazquez-Juarez et al., 2008), little is known of the role of G-protein coupled receptors in volume responses of cells to hypo- and hyperosmolar challenge. This finding may suggest that basal ion transport and secretory function of airway epithelium is not associated with muscarinic receptor control of volume.

There are few similarities between the effects of isosmolar solutions in the IPT *vis à vis* dispersed cells that tie cell shrinkage to EpDRF release. Whereas neither isosmolar D-M, NMDG-Glu nor urea elicited relaxation of the IPT, isosmolar NMDG-Glu caused extensive shrinkage in dispersed cells. In the IPT these agents elicited relaxation when applied in hyperosmolar concentrations either to MKH solution or in hyperosmolar jump maneuvers. Isosmolar NaCl, Na-Glu, and NMDG-Cl elicited relaxation in some preparations, but hyperosmolar additions of these agents always caused relaxation. Isosmolar NaCl had no effect, while NMDG-Cl, and Na-Glu elicited cell shrinkage in dispersed cells. Under isosmolar conditions, no K^+^ salt caused relaxation, whereas hyperosmolar concentrations of K^+^ salts did cause relaxation. On the other hand, isosmolar KCl and KBr caused swelling of dispersed epithelial cells.

In dispersed cells, isosmolar K^+^ salts gave rise to cell swelling or shrinkage, with a halide-dependence in the direction of the response: KGlu and K_2_SO_4_ evoked shrinkage, while KCl and KBr initiated swelling. The shrinkage caused by NMDG-Glu was greater than that caused by either NMDG-Cl or Na-Glu. A Na^+^-dependence of the volume response also was evident: NMDG-Glu caused greater shrinkage than Na-Glu. Despite the diversity of effects seen in the responses of dispersed cells to isosmolar solutions, the dissociation between the effects of isosmolar solutions on dispersed cells, where shrinkage was encountered, and adherent epithelium, where relaxation was largely not initiated by these solutions, is a second line of evidence that argues against the view that EpDRF release is initiated by cell shrinkage.

Differences between IPT and dispersed cells were also evident in the context of RVI. Isosmolar KCl and KBr, but not NaCl, increased cell volume, but neither condition initiated relaxation of IPT, and yet hyperosmolar jump caused rapid cell shrinkage followed by RVI. In contrast, IPT preparations remained relaxed as long as hyperosmolar conditions were present, either after hyperosmolar addition alone or hyperosmolar jump, suggesting that EpDRF release is a prolonged and not transient phenomenon. If RVI occurred in the adherent epithelium of the IPT, it was unrelated to the release of EpDRF.

Amiloride, DIDS and NPPB, but not bumetanide, inhibited hyperosmolar solution-induced relaxation of IPT and bioelectric responses (Fedan et al., 1999; Wu et al., 2004; Jing et al., 2008a). In unstimulated, dispersed cells, amiloride and bumetanide had little or no effect on cell volume, while DIDS (but not NPPB) reduced cell volume. Amiloride and DIDS inhibited modestly cell shrinkage in response to hyperosmolar challenge with D-M. DIDS had no effect on RVD in Calu-3 cells (Harron et al., 2009). Because Na^+^ and Cl^−^ transport are associated with EpDRF release and inhibit cell volume decrease, these findings are evidence that cell shrinkage could be required for EpDRF release. But in the face of other lines of evidence obtained in this study, this view is not tenable.

Confocal microscopy was utilized to evaluate the effects of hyperosmolar solutions on volume and *V*_*t*_ of adherent cells. Several differences between dispersed and adherent cells came to light as a result of these experiments: whereas as little as 10 mOsM increase induced shrinkage in dispersed cells, shrinkage and depolarization of adherent cells occurred at ≥120 mOsM D-M. That is, adherent cells are ~10–20-fold less sensitive to hyperosmolarity than dispersed cells. A second difference between the two preparations was neither isosmolar D-M nor isosmolar urea affected cell volume, and the subsequent addition of hyperosmolar D-M in the hyperosmolar jump protocol led to shrinkage, whereas the addition of hyperosmolar urea did not. Neither isosmolar D-M nor isosmolar urea evoked EpDRF-mediated relaxation responses in the IPT (Fedan et al., 2004a). In considering these findings and others in this study, it would appear that attachment to the airway wall influences the effects of isosmolar and hyperosmolar solutions on epithelial cell volume. The adherent cells apparently utilize basolateral ion transport to compensate so as to resist volume changes that would otherwise occur^1^. Nevertheless, low levels of hyperosmolarity trigger EpDRF release by mechanisms that involve changes in ion transport which do not affect cell volume. Freed of the basement membrane, dispersed cells now respond as non-polarized cells confronted with an altered osmolar milieu on their entire surface.

Cytoskeleton/microtubule-interfering inhibitors had no effect on relaxation responses of IPT to hyperosmolar challenge (Fedan et al., 2004b). In the present investigation five of these inhibitors evoked bioelectric responses of adherent cells involving both electrogenic (i.e., EHNA) and paracellular (i.e., latrunculin) pathways. Cytochalasin B alone affected *V*_*t*_ responses to MCh, which suggests that actin is somehow involved in muscarinic regulation of ion transport. Generally, none of the blockers affected bioelectric responses to D-M. These findings are in general agreement with those made in HL-60 cells (Hallows et al., 1991, 1996) but not PC12 cells (Fernandez and Pullarkat, 2010). In the absence of inhibitor effects on relaxation, and *V*_*t*_ and cell shrinkage responses, it would appear that structural elements in epithelium play little role in EpDRF release.

EHNA affected volume responses in dispersed cells, inhibiting responses to DMSO vehicle and decreasing the shrinkage response to hyperosmolar D-M. It is not known whether EHNA produced these effects by counteracting DMSO-stimulated increase in water permeability (Ellis et al., 1987; Zelenina and Brismar, 2000), redistributing of aquaporin 2 (Vossenkamper et al., 2007), or inhibiting dynein, phosphodiesterase 2 (Chambers et al., 2006) or adenosine deaminase.

Under conditions in which we have established that nystatin caused membrane permeabilization in the IPT, i.e., the short-circuited apical membrane revealed a basolateral Na^+^,K^+^-pump-driven *V*_*t*_ (Dodrill and Fedan, 2010), the drug caused hyperpolarization and potentiated MCh-induced *V*_*t*_ responses, whereas α-hemolysin did not. It is surprising that *V*_*t*_ responses to D-M were unaffected by either agent. In the IPT nystatin evoked contractions and potentiated relaxation responses to D-M, whereas α-hemolysin did not influence relaxation responses to hyperosmolar challenge (Fedan et al., 2004b).

We investigated the possibility that HICCs could be involved in responses of tracheal epithelial cells to hyperosmolar challenge because amiloride might inhibit hyperosmolar-induced relaxations of the IPT by an action at HICCs as well as at Na^+^ channels. Gadolinium had no effect itself on *V*_*t*_ or on responses to MCh, but it blocked the depolarizing responses to D-M. Inasmuch as these responses were also inhibited by amiloride, amiloride-sensitive HICCs appear to be involved in both types of responses. In contrast, flufenamic acid provoked a strong bioelectric response and did not influence D-M-induced *V_*t*_* responses. Collectively, these results suggest provisionally that the HICCs (Hoffmann et al., 2009; Koivusalo et al., 2009) are involved in the response to hyperosmolar challenge, and that the HICCs are of the type that are amiloride- and gadolinium-, but not flufenamic acid-, sensitive.

The depolarizing effect of flufenamic acid is of interest. Prevailing models of airway epithelial ion transport generally do not consider HICCs (Toczylowska-Maminska and Dolowy, 2012), although apical volume-sensitive outwardly-rectifying Cl^−^ channels (VSOC) that are sensitive to DIDS, NPPB and flufenamic acid and activated by swelling have been characterized in human airway epithelium (Okada et al., 2006; Toczylowska-Maminska and Dolowy, 2012). It is difficult to understand how the depolarization by flufenamic acid was mediated at the level of HICCs or VSOCs. A degree of promiscuity exists in its actions: serosal flufenamate depolarized colon, perhaps by inhibiting K^+^ conductance, Na^+^,K^+^-ATPase and cAMP-dependent Cl^−^ currents (Schultheiss et al., 2000).

In conclusion, adherent airway epithelial cells are very sensitive to small elevations in osmolarity at their apical surface and respond by releasing EpDRF, which relaxes airway smooth muscle. Dispersed airway epithelial cells also are very sensitive to the effects of raised osmolarity with an osmolar concentration-dependence which mimics that for EpDRF release, and respond with cell shrinkage. However, adherent epithelial cells are less sensitive to hyperosmolar solutions than dispersed cells in terms of the cell shrinkage response, with a concentration-dependence different from that of EpDRF release. The results buttress the earlier hypothesis that cell shrinkage *per se* is not a trigger of EpDRF release. The release of EpDRF by hyperosmolar solution is another role of airway epithelial cells: it serves to alter the function of airway smooth muscle.

### Conflict of interest statement

The authors declare that the research was conducted in the absence of any commercial or financial relationships that could be construed as a potential conflict of interest.

## Footnotes

^1^In separate unpublished studies, perfusion of the trachea with distilled water had no effect on epithelial cell morphology, i.e., did not cause cell lysis. However, dispersed cells placed in distilled water lysed immediately (not shown). Jumping from distilled water perfusion to hyperosmolarity in the IPT could not be recapitulated in dispersed cells, as they lysed when placed in distilled water.
